# An Analysis of Interactions between Fluorescently-Tagged Mutant and Wild-Type SOD1 in Intracellular Inclusions

**DOI:** 10.1371/journal.pone.0083981

**Published:** 2013-12-31

**Authors:** David A. Qualls, Keith Crosby, Hilda Brown, David R. Borchelt

**Affiliations:** Department of Neuroscience, Center for Translational Research in Neurodegenerative Disease, SantaFe HealthCare Alzheimer's Disease Research Center, McKnight Brain Institute, College of Medicine, University of Florida, Gainesville, Florida, United States of America; National Institute of Health, United States of America

## Abstract

**Background:**

By mechanisms yet to be discerned, the co-expression of high levels of wild-type human superoxide dismutase 1 (hSOD1) with variants of hSOD1 encoding mutations linked familial amyotrophic lateral sclerosis (fALS) hastens the onset of motor neuron degeneration in transgenic mice. Although it is known that spinal cords of paralyzed mice accumulate detergent insoluble forms of WT hSOD1 along with mutant hSOD1, it has been difficult to determine whether there is co-deposition of the proteins in inclusion structures.

**Methodology/Principal Findings:**

In the present study, we use cell culture models of mutant SOD1 aggregation, focusing on the A4V, G37R, and G85R variants, to examine interactions between WT-hSOD1 and misfolded mutant SOD1. In these studies, we fuse WT and mutant proteins to either yellow or red fluorescent protein so that the two proteins can be distinguished within inclusions structures.

**Conclusions/Significance:**

Although the interpretation of the data is not entirely straightforward because we have strong evidence that the nature of the fused fluorophores affects the organization of the inclusions that form, our data are most consistent with the idea that normal dimeric WT-hSOD1 does not readily interact with misfolded forms of mutant hSOD1. We also demonstrate the monomerization of WT-hSOD1 by experimental mutation does induce the protein to aggregate, although such monomerization may enable interactions with misfolded mutant SOD1. Our data suggest that WT-hSOD1 is not prone to become intimately associated with misfolded mutant hSOD1 within intracellular inclusions that can be generated in cultured cells.

## Introduction

Mutations in the gene encoding superoxide dismutase 1 (SOD1) cause ∼20% of the cases of familial amyotrophic lateral sclerosis (fALS). SOD1 is a relatively small enzyme comprised of 153 amino acids; in its active state the protein homodimerizes to form the mature enzyme with each subunit binding 1 atom of Zn and 1 atom of Cu [1]. To date more than 165 mutations in more than 75 positions, in the enzyme, have been identified in patients diagnosed with ALS (http://alsod.iop.kcl.ac.uk/Als/Index.aspx). Initial work to characterize the impact of disease causing mutations on the biology of SOD1 demonstrated that interactions between the normal and mutant proteins occurred [2], but the role of such interactions in disease pathogenesis was uncertain. One common feature of mutant SOD1 proteins is that they exhibit a high tendency to aggregate into high molecular weight structures that are insoluble in non-ionic detergents [3].

To study interactions between WT and misfolded mutant SOD1, we have previously used a strategy in which SOD1 is fused in frame to either red fluorescent protein (turbo RFP) or yellow fluorescent protein (YFP) [4]. By this method, we can visualize the misfolding of mutant SOD1 in the formation of inclusion-like structures [4]. Fusions of SOD1 to eGFP have been shown to produce proteins in which SOD1 dimeric interactions occur, and the enzyme retains activity [5]. In the present study, we present a comprehensive assessment of interactions between WT and mutant human hSOD1 proteins in culture cell models of aggregation. Our findings indicate that such interactions can be influenced by the nature of the fluorophore tag. In general, the data involving WT hSOD1 fused with YFP were the least complicated to interpret. The weight of evidence from our studies argues that, within the short time-frame of mutant SOD1 aggregation that is modeled in cultured cells, WT-SOD1 does not readily interact with misfolded mutant SOD1 within cytosolic inclusions.

## Methods

### DNA expression plasmids

Expression plasmids that encode wild-type (WT), A4VSOD1, and G37RSOD1 fused to RFP and YFP have been previously described [4], [6]. These original constructs were generated from an SOD1:YFP fusion protein cDNA (pPD30.38) that was kindly provided by Dr. Rick Morimoto (Northwestern University). This SOD1::eYFP construct contained a 27 bp linker (translated sequence—LQLKLQASA) between SOD1 and YFP that we modified to include a Sal1 restriction site (new translated linker sequence—LQSTLQASA). Our modified SOD1:YFP DNA fusion construct was then cloned into the mammalian pEF-BOS expression vector [7]. From this initial SOD1:YFP expression plasmid, we generated vectors for A4V-hSOD1:YFP and G37R-hSOD1:YFP by cloning in PCR amplified cDNA from pre-existing pEF.BOS vectors [3], [8], [9], utilizing an Nco 1 site at the 5′ end of the open reading frame and introducing a Sal 1 site at the 3′ end of the open reading frame in a manner that eliminated the stop codon and allowed for joining the SOD1 cDNA in-frame with YFP [4]. A similar approach was used to create SOD1 fusion proteins with RFP [Turbo RFP cDNA obtained from the pTRIPZ empty vector available at Open Biosystems (Huntsville, AL, USA)] by replacing the YFP tag with the RFP tag. In this way, we created WT-hSOD1:RFP, A4V-hSOD1:RFP and G37R-hSOD1:RFP constructs. For the present study, additional constructs were created by replacing the SOD1 portion of these previously made constructs with PCR amplified cDNAs for the human SOD1-FG50/51EE (engineered monomer [10], [11]; abbreviated hWTSOD1mon) or G85R-hSOD1.

### Cell transfections

For cell transfection studies, we used Chinese Hamster Ovary (CHO) cells because these cells normally show a very flat morphology with a distinct nucleus and cytoplasm; allowing for a good visualization of intracellular inclusions. These cells also show good adherence to culture plates and resist lifting after saponin treatment. Cells were split into 12-well plates containing Poly-L-Lysine coated coverslips, and incubated at 37°C with 5% CO2 for 24 hours. Cells were transiently transfected with the vectors of interest using Lipofectamine-2000 (single transfections: 500 ng total DNA used; co-transfections: 500 ng of each construct used). 24 hours after transfection, one set of cells were treated with 0.1% saponin (Fluka/Sigma-Aldrich, St. Louis, Mo) in PBS for 30 minutes. The cells were then rinsed with PBS and fixed in 4% paraformaldehyde in PBS. A 1∶2000 solution of DAPI in PBS was used to stain nuclei. Coverslips were then mounted on slides for analysis via fluorescence microscopy.

All single and co-transfections were performed three times. Each sample was analyzed for the presence and composition of inclusion-like structures. Representative examples of cells from each sample were photographed. The camera exposures used to capture RFP and YFP images in co-transfections were recorded and compared to single transfections to ensure that the fluorescence from YFP was the result of the intended fluorescent protein rather than bleed-through from co-expressed RFP.

## Results

### Visualization of WT and mutant SOD1 interactions in the formation of intracellular inclusions

**Figure 1:**
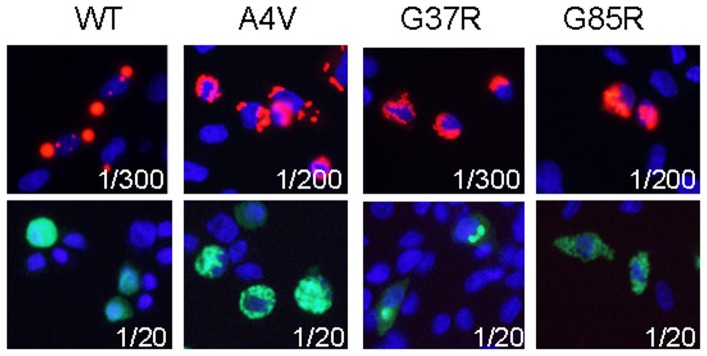
Mutant SOD1 fused to either RFP or YFP forms inclusions with similar morphologies. CHO cells were transiently transfected with vectors to expression WT and ALS-associated variants (A4V, G37R, G85R). After 24 hours, the cells were fixed in paraformaldehyde and imaged. The exposure times are noted on the images. WT-hSOD1:RFP produces round, well defined inclusions. WT-hSOD1:YFP diffusely fills the cytosol (rounded cell in the image shown). Mutant SOD1 fused to either RFP or YFP form variegated perinuclear inclusions. The images shown are representative of 3 independent transfection experiments, analyzing between 200 and 1,000 individual cells.

To examine interactions between WT and mutant human SOD1 (hSOD1) in the formation of aberrant aggregate inclusions, we used a strategy in which variants of hSOD1 were fused to either RFP or YFP following a previously described approach [4]. As previously described when these proteins were expressed in HEK293FT cells[4], [6], when expressed in CHO cells fusion proteins of WT-hSOD1 to RFP (WT-hSOD1:RFP) formed large well delineated cytoplasmic inclusions whereas WT-hSOD1 fused to YFP did not form such inclusions but instead filled the cell with diffusely distributed fluorescence (Fig. 1). Fusions of RFP or YFP with mutant hSOD1 (A4V, G37R, or G85R) produced inclusions that were morphologically distinct from those of the WT-SOD1:RFP proteins (Fig. 1). Inclusions formed by YFP and RFP fusions to mutant hSOD1 could be described as perinuclear ring-like or multi-focal structures; we referred to these structures as possessing a variegated morphology (Table S1).

**Figure 2:**
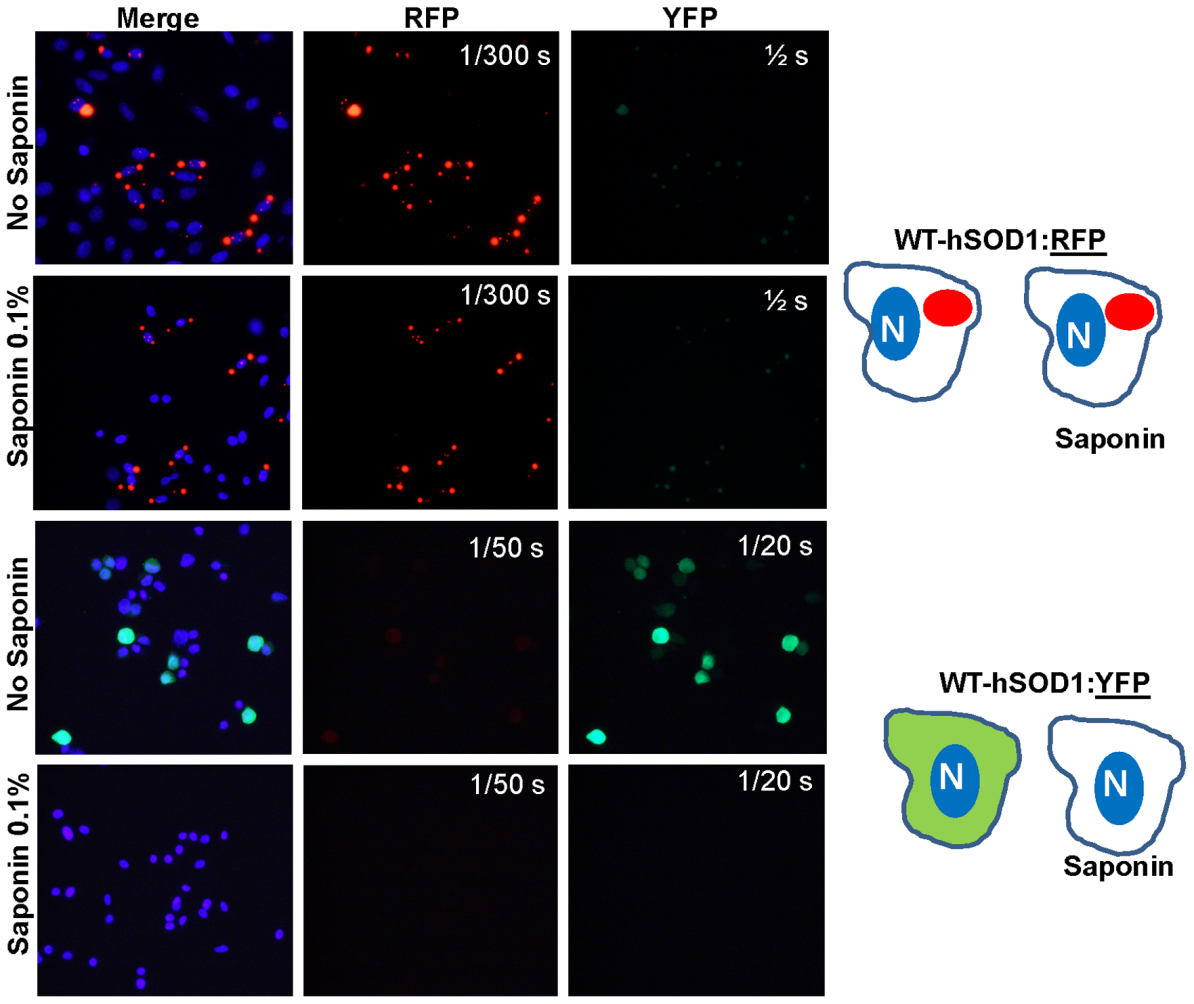
Inclusions formed by WT-hSOD1:RFP are not released by saponin. CHO cells were transiently transfected with expression vectors for the two SOD1 constructs shown. After 24-hSOD1:YFP is fully releasable by saponin treatment whereas WT-hSOD1:RFP remained cell-associated. The images shown are representative of 3 independent transfection experiments, analyzing between 200 and 1,000 individual cells.

**Figure 3:**
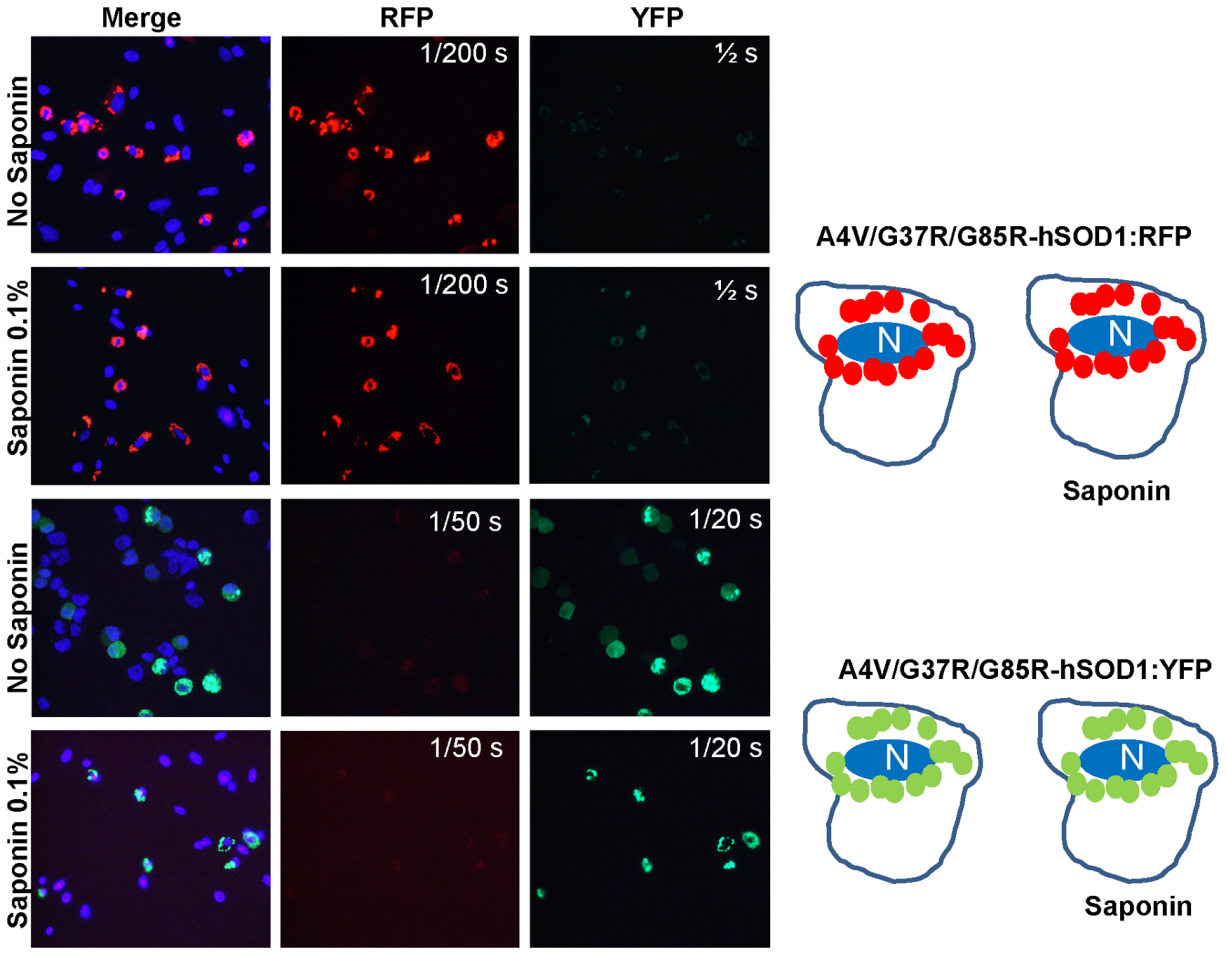
Mutant SOD1 fused to RFP or YFP form similar types of inclusions that resist release by saponin. CHO cells were transiently transfected with expression vectors for A4V-hSOD1:RFP or A4V-hSOD1:YFP. After 24 hours the cells were treated, or not, with saponin, fixed in paraformaldehyde, and imaged. Inclusions formed by mutant hSOD1 fused to either RFP or YFP remained cell-associated after saponin treatment. The images shown are representative of 3 independent transfection experiments, analyzing between 200 and 1,000 individual cells. Similar observations were made with cells expressing G37R or G85R hSOD1 fused to either RFP or YFP (see Figures S1and S2).

In a recent study, we demonstrated that we can further distinguish aggregated SOD1 from soluble protein by treating cells with saponin (an amphipathic glycoside that creates holes in the plasma membrane without lysing the cell; for review see [12]). In all of the experiments that follow, experiments were performed in pairs in which one culture was treated with saponin before immunostaining, following a previously published paradigm [6]. Similar to what we previously reported for mutant SOD1 fusions with YFP [6], the inclusions formed by WT-hSOD1:RFP were found to remain cell associated after treatment with saponin (Fig. 2). As previously reported [6], WT-hSOD1-YFP fusions proteins were completely released by saponin treatment (Fig. 2) whereas mutant hSOD1 fusions to either RFP or YFP formed variegated inclusion-like structures that remained cell-associated after saponin treatment (Fig. 3 example of A4V-hSOD1 fused to RFP or YFP; Figs. S1 and S2 show data for G37R and G85R SOD1 variants).

Importantly, the RFP protein was much brighter than the YFP protein and thus the exposure times were adjusted to capture the images at equivalent intensities. Typically, images of RFP fluorescence were captured with exposures of 1/200 to 1/300 sec whereas exposures of YFP fluorescence were 1/20 to 1/30 sec (Fig. 3). We observed that exposure times of up to ½ to ⅓ sec in the YFP channel were possible for cells expressing RFP fusions, but at these lengths of exposure some minimal bleed-through of RFP into the YFP channel was noted (Fig. 3, see YFP image in row 2). Thus, in experiments in which RFP and YFP fusion proteins were co-transfected to observe co-localization, weak signals in the YFP channel upon long exposure should be viewed with the caveat that some weak bleed-through of very bright RFP structures was possible.

### Analysis of interactions between WT and mutant human SOD1

In all of the observations that are described below, the outcomes essentially were largely all or none; meaning that if one of the expressed RFP tagged SOD1 variants formed an inclusion, then most inclusions also contained the YFP protein or none contained it. Similarly if one of the YFP tagged variants of SOD1 formed an inclusion, then most also contained the RFP tagged protein or none contained it. Thus, the data were analyzed for morphological outcomes in assessing whether or not the YFP and RFP tagged proteins produced inclusions, whether SOD1 variants fused to these fluorescent proteins co-localized in co-transfection experiments, and whether each of the fluorescent fusion proteins was resistant to saponin.

**Figure 4:**
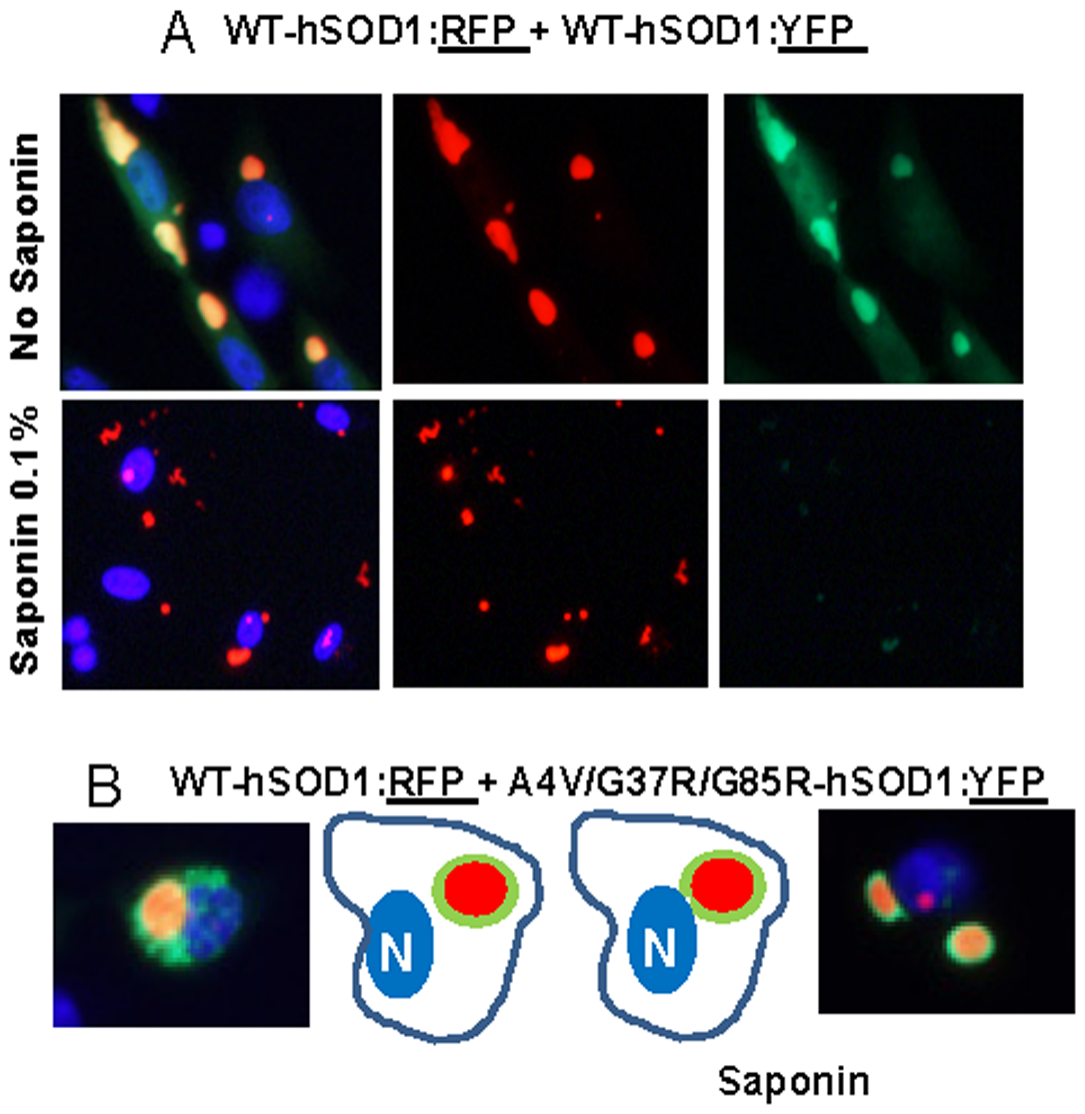
Co-expression of WT-hSOD1:RFP with WT and mutant SOD1 fused to YFP. CHO cells were transiently transfected with expression vectors for the SOD1 constructs shown. After 24-hSOD1:RFP forms well defined round inclusions that are not released by saponin. Co-expressed WT-hSOD1:YFP appears to be closely associated with these inclusions, but after saponin this protein is released whereas the WT-hSOD1:RFP remains cell associated. B, Mutant SOD1:YFP appears to be more tightly bound to the surface of inclusions formed by WT-hSOD1:RFP. At least three independent transfection experiments were performed and between 200 and 1,000 individual cells were analyzed in compiling these data.

In a prior study, we had investigated interactions between WT-hSOD1:RFP and WTh-SOD1 fused to YFP; observing that it appeared that WT-hSOD1:YFP was intimately associated with the large round inclusions formed by WT-hSOD1:RFP [4], [6]. However, we now observed that the co-expressed WT-hSOD1:YFP was released by saponin; whereas WT-hSOD1:RFP inclusions remained behind (Fig. 4A). By contrast, mutant fusion proteins of SOD1:YFP remained associated with the WT-hSOD1:RFP inclusion after saponin; appearing to be deposited on the surface of the WT-hSOD1:RFP structure (Fig. 4B; Fig. S3 and S4). This initial finding suggested that WT-hSOD1 could potentially interact with misfolded mutant SOD1.

**Figure 5:**
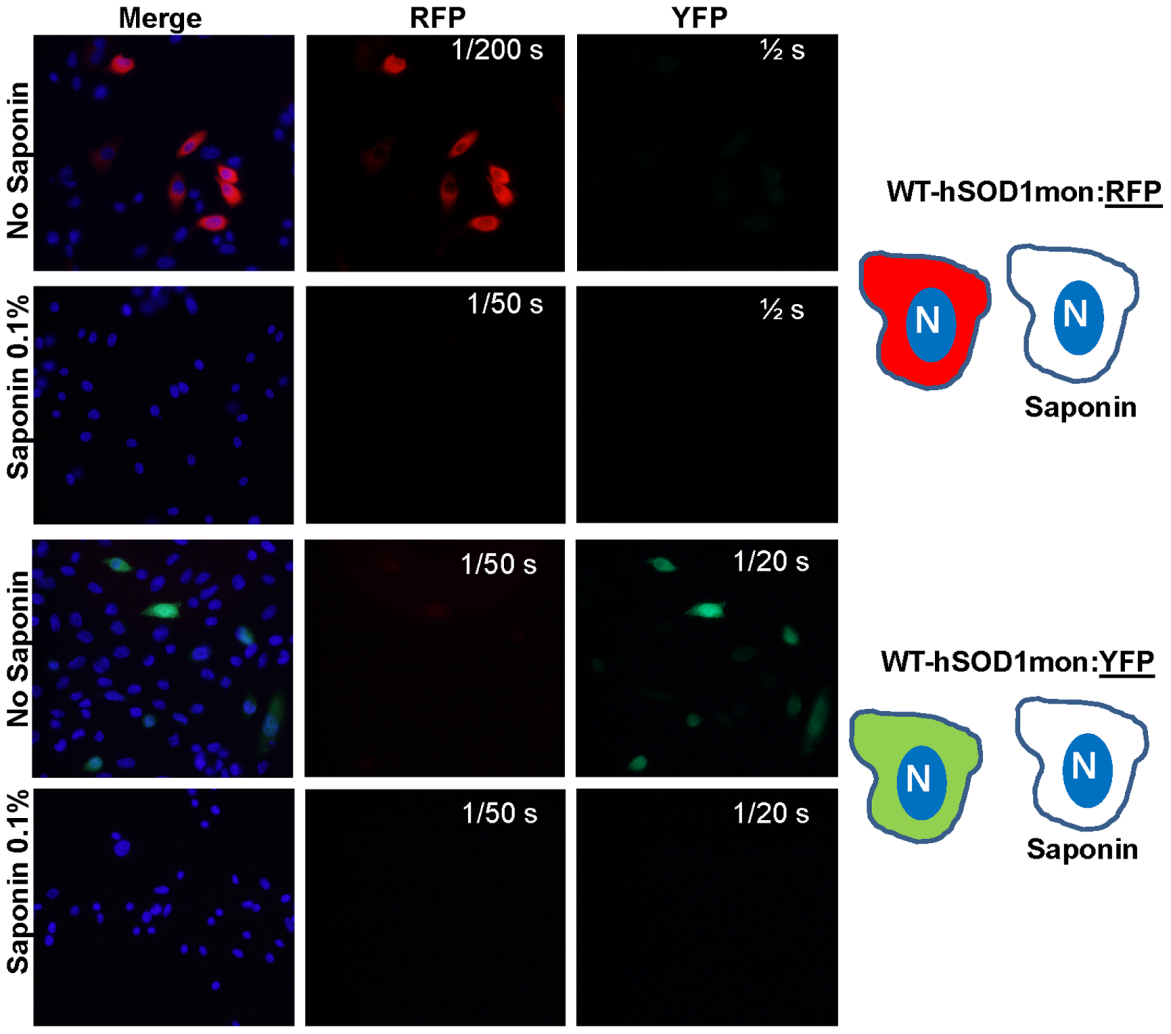
Experimental monomerization of WT-hSOD1 does not induce inclusion formation. Variants of WT-hSOD1 encoding mutations at amino acids 50/51 that monomerize the proteins were fused to RFP or YFP. In transiently transfected CHO cells, both variants exhibit a diffuse distribution in the cell and remain solubilizable by saponin. The images shown are representative of 3 independent transfection experiments, analyzing between 200 and 1,000 individual cells.

**Figure 6:**
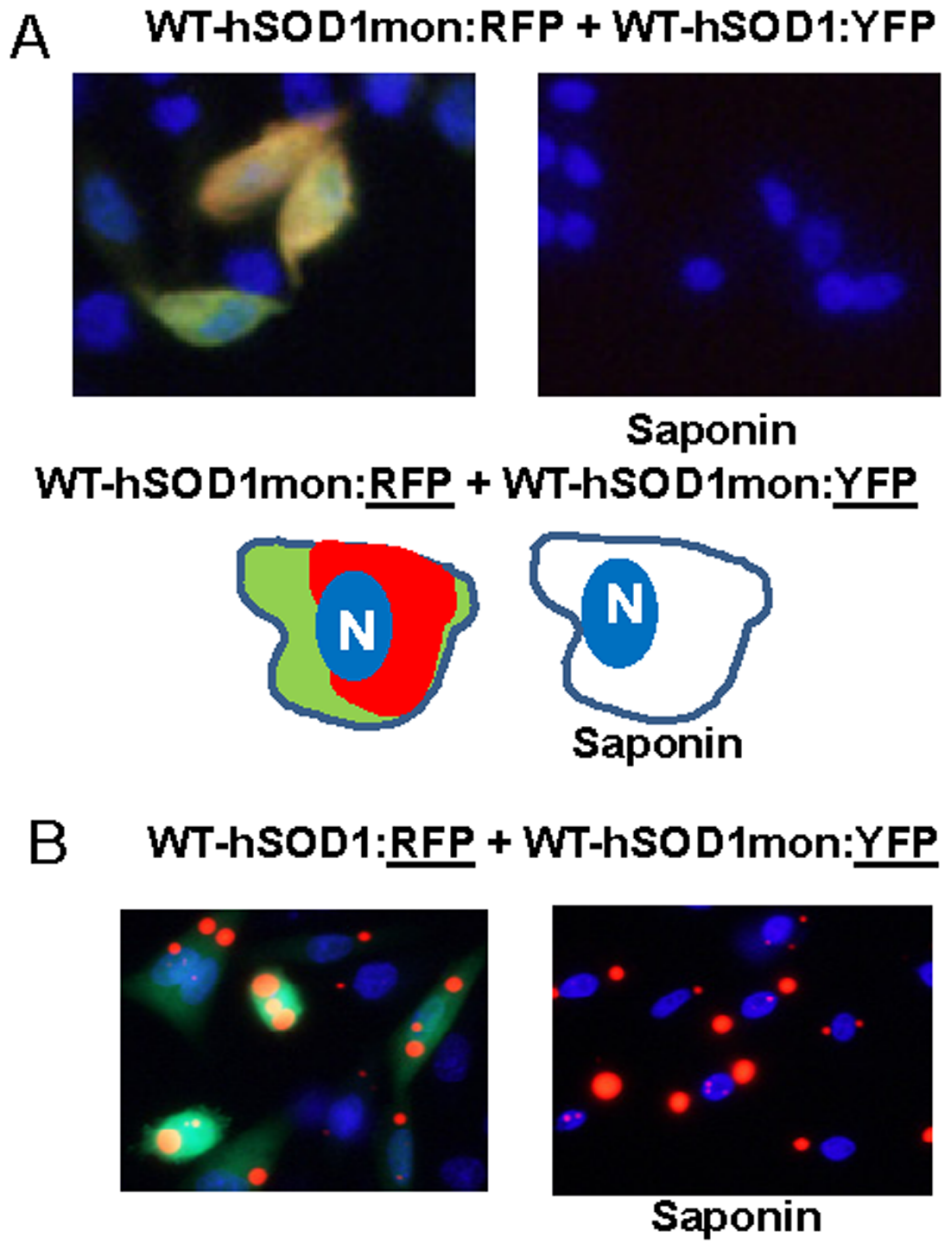
Co-expression of WT-hSOD1mon:RFP with WT-hSOD1 and WT-hSOD1mon fused to YFP. CHO cells were transiently transfected with expression vectors for the SOD1 constructs shown. After 24-expression of WT-hSOD1:RFP with either WT-hSOD1:YFP or WT-hSOD1mon:YFP does not produce inclusions; all proteins remain soluble in saponin. B, WT-hSOD1:RFP co-expressed with WT-hSOD1mon:YFP demonstrates a lack of tight binding between these proteins. At least three independent transfection experiments were performed and between 200 and 1,000 individual cells were analyzed in compiling these data.

An important feature of the version of RFP that was used for these constructs is that it is known to dimerize whereas YFP is primarily monomeric [13]. Thus, the WT-SOD1:RFP fusion protein was essentially a bivalent molecule in which each entity in the fusion protein could independently dimerize with its respective partner. To determine how this bivalency influenced the ability of WT-SOD1:RFP to form inclusions, we fused the monomeric variant of WT-SOD1 (SOD1-F50E/G51E; [10], [11] to RFP (WT-hSOD1mon:RFP) and YFP (WT-hSOD1mon:YFP). When expressed at high levels in CHO cells, we found the WT-hSOD1mon fusions to RFP or YFP remained soluble and completely releasable by saponin (Fig. 5). Co-expression of WT-hSOD1mon:RFP with WT-hSOD1:YFP (Fig. 6A; Fig. S5) or WT-hSOD1mon:RFP with WT-hSOD1mon:YFP (Fig. S5) did not induce inclusions and both proteins remained soluble in saponin (Table S2). Similar to WT-hSOD:YFP (see Fig. 4A), WT-hSOD1mon:YFP did not bind tightly to inclusions formed by WT-hSOD1:RFP (Fig. 6B; Fig. S6; Table S2). Collectively, these data suggested that the mutations to monomerize WT-hSOD1 did not induce the protein to form inclusion aggregates.

**Figure 7:**
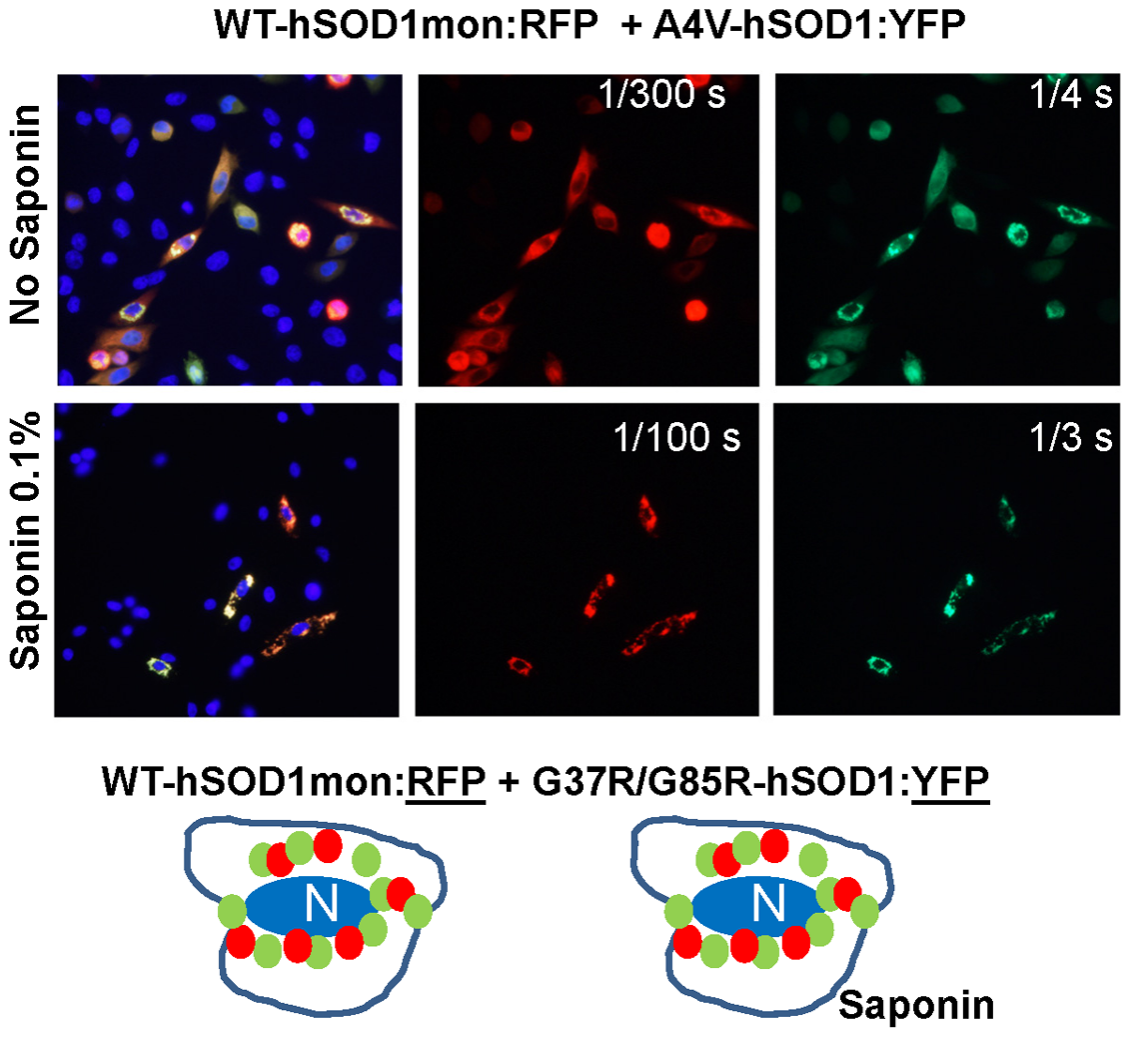
Co-expression of WT-hSOD1mon:RFP with mutant hSOD1 fused to YFP. Representative image of cells co-expressing WT-hSOD1mon:RFP and A4V-hSOD1:YFP. Images showing cell co-expressing WT-hSOD1mon:RFP with G37R- or G85R-hSOD1:YFP are provided in Figures S7 and S8. Cells were fixed and imaged 24 hours post-transfection with or without prior treatment with saponin. At least three independent transfection experiments were performed and between 200 and 1,000 individual cells were analyzed in compiling these data.

To determine the role of normal dimeric interactions between WT and mutant SOD1 in the formation of mixed aggregates, we performed a series of experiments in which plasmids encoding mutant hSOD1 fused to YFP (A4V, G37R, G85R) were co-transfected with plasmids encoding hWTmon-RFP. In these combinations, the WT-hSOD1mon:RFP adopted the more variegated inclusion morphology of A4V-hSOD1:YFP structures with both proteins exhibiting resistance to saponin (Fig. 7; and Figs. S7 and S8 for examples of WT-hSOD1mon:RFP co-expressed with G37R and G85R-hSOD1 fused to YFP) (Table S2). These findings suggested that monomerization of WT-hSOD1 could promote an integral interaction with misfolded mutant hSOD1.

**Figure 8:**
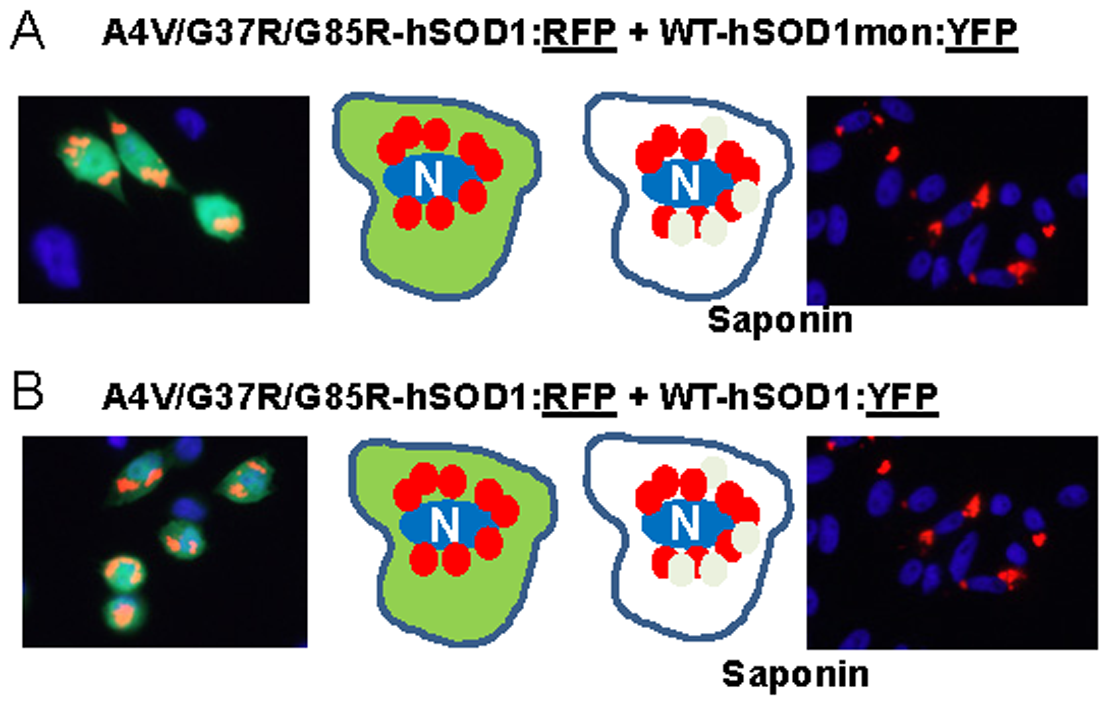
Co-expression of mutant hSOD1:RFP with WT-hSOD1mon:YFP or WT-hSOD1:YFP. CHO cells were transiently transfected with expression vectors for the SOD1 constructs shown. After 24:RFP produces inclusions that only weakly bind WT-hSOD1mon:YFP or WT-hSOD1:YFP. At least three independent transfection experiments were performed and between 200 and 1,000 individual cells were analyzed in compiling these data.

**Figure 9:**
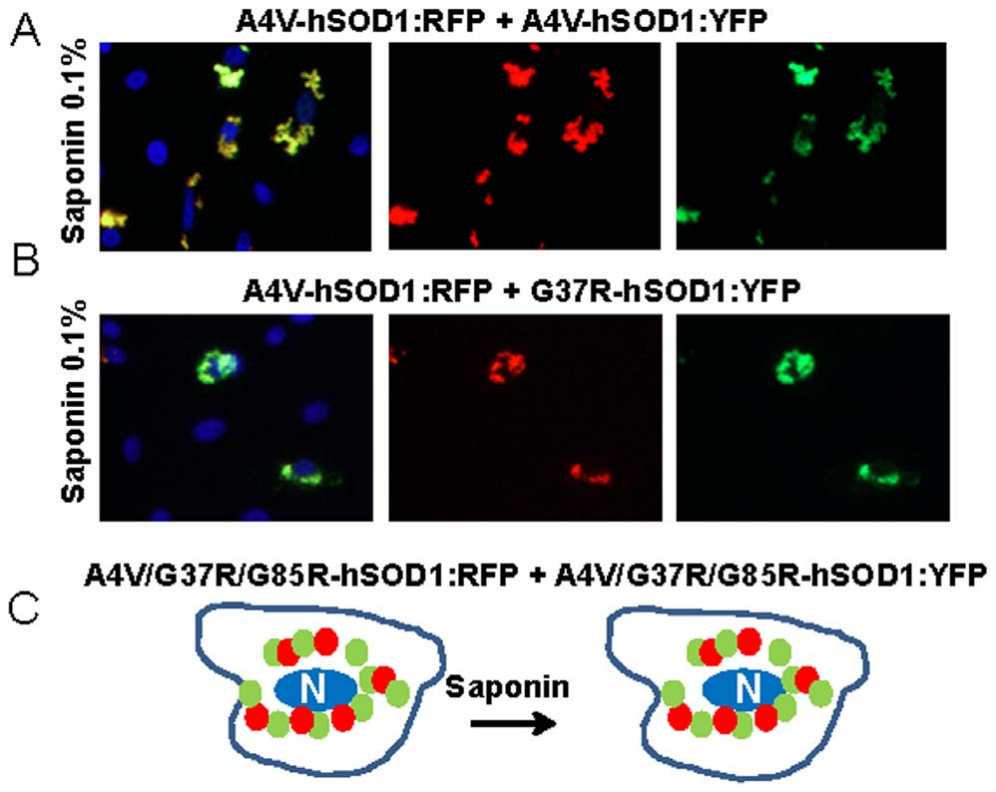
Co-expression of mutant hSOD1 fused to RFP with mutant hSOD1 fused to YFP. In a matrix approach, all possible combinations for the 6 fusion constructs of mutant SOD1 fused to RFP or YFP were examined. In all cases, inclusions contained both proteins in saponin-resistant aggregates. At least three independent transfection experiments were performed and between 200 and 1,000 individual cells were analyzed in compiling these data.

In experiments in which we reversed the fluorescent tags such that mutant hSOD1 proteins were fused to RFP (A4V, G37R, and G85R) and the WT-hSOD1mon or WT-hSOD1 proteins were fused to YFP, then we observed less robust interactions. When mutant hSOD1:RFP (A4V, G37R, and G85R) was co-expressed with WT-hSOD1mon:YFP (Fig.8A and Figs. S9–S11) (Table S3), or when co-expressed with WT-hSOD1:YFP (Fig. 8B and Figs. S12–S14) (Table S3) the YFP fusion proteins remained fully releasable by saponin. For comparison, when mutant hSOD1:RFP fusions were co-expressed with mutant hSOD1:YFP fusions, we observed completely intermingled aggregates that were resistant to saponin regardless of whether the two fluorophores were fused to the same mutant or to different mutants (Fig. 9; and Figs. S15–S20 for examples of all combinations) (Table S4). Thus, it seemed that when mutant SOD1:RFP was co-expressed with WT or WT-SOD1mon YFP fusion proteins, the two WT:YFP variants interacted only weakly with mutant SOD1:RFP inclusions. By contrast, the co-mingling of inclusions formed by mutant SOD1 fused to YFP with mutant SOD1 fused to RFP indicated that the two fluorophores were compatible; that is they did not prevent inclusion formation. Thus the lack of a tight association between WT-hSOD1, or WT-hSOD1mon, with inclusions formed by mutant SOD1 fused to RFP could be interpreted as evidence that WT-hSOD1 and monomeric hSOD1 are not inherently prone to interact with misfolded mutant SOD1 within inclusions.

## Discussion

In the present study, we describe a comprehensive assessment of the behavior of WT and mutant SOD1 fused to RFP and YFP fluorophores (Table 1). A significant methodological finding was that the nature of the fluorophore directly impacted the behavior of the protein. Despite this problem, there were some consistent observations. 1) SOD1 encoding mutations linked fALS and fused to either RFP or YFP produced inclusion like structures that do not readily diffuse out of permeabilized cells. 2) Monomerizing mutations in SOD1 do not induce inclusion formation. 3) SOD1 proteins encoding different fALS mutations can readily form intermingled inclusions containing both proteins. The less consistent outcomes involved examinations of interactions between WT and mutant SOD1. WT-hSOD1:YFP fusion proteins failed to show strong interactions with misfolded mutant SOD1:RFP within inclusions. However, WT-hSOD1:RFP, which formed large round inclusions on its own, appeared to co-aggregate with mutant SOD1:YPF concentrated at the margin of the RFP containing structure. We could accept the argument that the apparent interaction between WT-hSOD1:RFP and misfolded mutant SOD1 fused to YFP is indicative that the potential does exist for WT and mutant SOD1 to interact in the formation of inclusions. However, we view the combinations of mutant SOD1 fused to RFP with WT SOD1 fused to YFP as being more informative as to how soluble WT-hSOD1 may behave in the presence of an aggregating mutant SOD1 protein.

**Table 1:** Matrix table to summarize morphology of inclusions in cells expressing RFP and YFP tagged variants of SOD1.

Co-transfected construct	none	WT-hSOD1:YFP	WT-hSOD1mon:YFP	A4V-hSOD1:YFP	G37R-hSOD1:YPF	G85R-hSOD1:YFP
none		No inclusions	No inclusions	Variegated saponin resistant inclusions		
WT-hSOD1:RFP	Round saponin resistant inclusions	Round intermingled inclusions only RFP inclusions are saponin resistant	Round RFP only inclusions. Only RFP inclusions are saponin resistant	Round inclusions with the YFP fusion appearing to be layered on the surface of the RFP structure. Both RFP and YFP fusion proteins in these inclusions are saponin resistant		
WT-hSOD1mon:RFP	No inclusions	No inclusions	No inclusions	Variegated intermingled inclusions; both RFP and YFP inclusions are saponin resistant		
A4V-hSOD1:RFP	Variegated saponin resistant inclusions	RFP variegated inclusions; only RFP inclusions are saponin resistant	RFP variegated inclusions only RFP is saponin resistant			
G37R-hSOD1:RFP						
G85R-hSOD1:RFP						

In previous studies, we have used approaches similar to what were used here to examine interactions between WT and mutant hSOD1 [4]. In the experimental evolution of our work on SOD1 aggregation in cell culture models, we observed that we could readily distinguish soluble SOD1 (whether fused to a fluorescent tag or not) from insoluble aggregated SOD1 by treatment of the cells with saponin [6]. This molecule interacts with cholesterol to produce pores in the plasma membrane that allow soluble and readily diffuse-able proteins to release into the aqueous medium [14], [15]. Thus, saponin treatment allowed us to more rigorously determine whether WT SOD1 is tightly associated with mutant SOD1 in aggregates.

From previous work, we knew that expression of a fusion of mutant hSOD1 to RFP in cultured cells produced inclusions whereas fusion of WT-hSOD1 to YFP produced a soluble protein [6]. In prior work, when mutant hSOD1:RFP was co-expressed with WT-hSOD1:YFP, we observed the two proteins closely associated in inclusion-like structures [6]. In the present study, we now show that the WT-hSOD1:YFP that seemed to be associated with the mutant SOD1 inclusion largely dissociates with saponin treatment. In co-transfections of WT-hSOD1:RFP with WT-hSOD1mon:YFP, the YFP signal remained largely diffuse and was easily released into medium by saponin. These data indicate that the inclusions formed by mutant-hSOD1:RFP leave the SOD1 component of the protein unavailable for pairing with either native or monomerized WT-hSOD1 within the YFP fusion protein.

The observation that WT-SOD1:RFP forms inclusions and that monomerization of the protein by mutation converts the protein to a soluble molecule has implications in our interpretation of data derived from mutant hSOD1 fused to RFP. The RFP molecule is known to dimerize and thus the WT-SOD1:RFP proteins possess two elements that dimerize; the SOD1 domain and the RFP domain [13]. Experimental conversion of SOD1 from a dimeric to a monomeric enzyme by the mutation of residues 50 and 51 from FG to EE was first described by Bertini et al [10]. It is thought that the introduction of charged residues at these sites produces a repulsive effect as the two monomers of SOD1 attempt to align as a homodimeric enzyme [10], [11]. These monomeric enzymes retain activity and crystal structures of this experimental variant have demonstrated that the monomeric proteins fold into a near normal conformation [11]. Thus, the engineered monomer of SOD1 is thought to be WT-like in its properties. Our observation that hWTmon:RFP proteins remain fully soluble suggests to us that the formation of aggregates by WT-SOD1:RFP may be occurring by a process that is unrelated to SOD1 misfolding but rather potentially due to the formation of interconnected networks between what are essentially bivalent proteins.

Although the RFP tag clearly altered the behavior of WT-hSOD1, it is less certain as to whether the tag influenced the behavior of mutant hSOD1. All three of the hSOD1 mutants we fused to RFP probably retain the ability to homodimerize and thus inclusions formed by A4V, G37R, or G85R-hSOD1 fused to RFP could also include bivalent interactions similar to what we propose for WT-hSOD1:RFP inclusion. However, morphologically, WT-hSOD1:RFP inclusions were distinct from the inclusions produced by mutant hSOD1:RFP fusions; and additionally, the morphology of the mutant hSOD1:YFP fusions (YFP is monomeric [13]) matched that of the mutant hSOD1:RFP fusions. We also observed that co-expression of different mutant hSOD1 variants fused to RFP and YPF (e.g. A4V-hSOD1:RFP with G37R-hSOD1:YFP) produced completely comingled inclusions for every possible combination. Collectively, these observations suggest that the RFP tag exerted little if any impact on the misfolding of mutant SOD1. Thus, we are inclined to conclude that mutant SOD1 tagged with RFP is a useful reporter and thus we view the failure of WT-hSOD1:YFP to interact with inclusions formed by these RFP tagged proteins as highly suggestive evidence that WT-hSOD1 is not very prone to co-aggregate with mutant SOD1.

For the monomeric variants of WT-hSOD1, the picture is more complicated. Although monomeric hSOD1 did not readily aggregate, WT-hSOD1mon:RFP was capable of fully co-mingling with mutant SOD1:YFP proteins in saponin resistant inclusions. Notably, the monomeric variants of WT-hSOD1 behaved as fully soluble proteins whether fused to RFP or YFP. On face value, the data indicate that monomeric WT-hSOD1 can more readily interact with misfolded mutant SOD1 in the formation of inclusions. However, we cannot be certain of this conclusion because the supporting data draw heavily on the behavior of the RFP fusion proteins. Importantly, we observed that neither WT-hSOD1:YFP nor WT-hSOD1mon:YFP associated with the inclusions formed by mutant SOD1:RFP fusion proteins in a saponin-resistant manner. The lack of agreement between these sets of experiments complicates interpretation of the data as to whether monomerization of WT SOD1 facilitates an association with mutant SOD1 in inclusions. In one condition we see an association, but the effect was inconsistent.

## Conclusions

Our findings clearly show that fluorescent proteins tags that are commonly used to track the behavior of proteins in living cells are not completely benign markers. That said; our data indicate that YFP is probably less intrusive than RFP. In this comprehensive set of experiments in which we have performed all combinations of tagging, we find several consistent features. First, mutant SOD1 fusion to either RFP or YFP produced inclusion-like structures. Second, experimental mutations that monomerize SOD1 do not heighten its propensity to form inclusions. Third, SOD1 proteins encoding different fALS mutations can readily form intermingled inclusions containing both proteins. Because WT-hSOD1 fused to RFP formed inclusions on its own, we do not view the association of this protein with inclusions formed by mutant SOD1 fused to YFP as being informative. Instead we are inclined to place greater weight on the studies in which mutant SOD1 fused to RFP was co-transfected with WT-hSOD1 fused to YFP. If we focus on these data, it appears that in our cultured cell models of aggregation WT-hSOD1 is not highly prone to interact with misfolded mutant SOD1 in the formation of inclusions. Additionally, mutations that monomerize WT-hSOD1 do not consistently promote interaction with mutant SOD1 in inclusions. From these data, we predict that WT-hSOD1 may be relatively slow to interact with misfolded mutant SOD1. The much longer timelines of mutant SOD1 misfolding and aggregation that occur *in vivo*, however, clearly changes the dynamics of what could happen.

## Supporting Information

Figure S1 Representative images from cells expressing G37R-hSOD1:RFP or G37R-hSOD1:YFP.
(PDF)

Figure S2 Representative images from cells expressing G85RR-hSOD1:RFP or G85R-hSOD1:YFP.
(PDF)

Figure S3 Representative images from cells co-expressing WT-hSOD1:RFP and A4V-hSOD1:YFP; and cells co-expressing WT-hSOD1:RFP and G37R-hSOD1:YFP.
(PDF)

Figure S4 Representative images from cells co-expressing WT-hSOD1:RFP and G85R-hSOD1:YFP.
(PDF)

Figure S5 Representative images from cells co-expressing WT-hSOD1mon:RFP and WT-hSOD1:YFP; and cells co-expressing WT-hSOD1mon:RFP and WT-hSOD1mon:YFP.
(PDF)

Figure S6 Representative images of cells co-expressing WT-hSOD1:RFP and WT-hSOD1mon:YFP.
(PDF)

Figure S7 Representative images from cells co-expressing WT-hSOD1mon:RFP and G37R-hSOD1:YFP.
(PDF)

Figure S8 Representative images from cells co-expressing WT-hSOD1mon:RFP and G85R-hSOD1:YFP.
(PDF)

Figure S9 Representative images from cells co-expressing A4V-hSOD1:RFP and WT-hSOD1mon:YFP.
(PDF)

Figure S10 Representative images from cells co-expressing G37R-hSOD1:RFP and WT-hSOD1mon:YFP.
(PDF)

Figure S11 Representative images from cells co-expressing G85R-hSOD1:RFP and WT-hSOD1mon:YFP.
(PDF)

Figure S12 Representative images from cells co-expressing A4V-hSOD1:RFP and WT-hSOD1:YFP.
(PDF)

Figure S13 Representative images from cells co-expressing G37R-hSOD1:RFP and WT-hSOD1:YFP.
(PDF)

Figure S14 Representative images from cells co-expressing G85R-hSOD1:RFP and WT-hSOD1:YFP.
(PDF)

Figure S15 Representative images from cells co-expressing A4V-hSOD1:RFP and A4V-hSOD1:YFP or G37R:hSOD1:YFP.
(PDF)

Figure S16 Representative images from cells co-expressing A4V-hSOD1:RFP and G85R:hSOD1:YFP.
(PDF)

Figure S17 Representative images from cells co-expressing G37R-hSOD1:RFP and A4V-hSOD1:YFP or G37R-hSOD1:YFP.
(PDF)

Figure S18 Representative images from cells co-expressing G37R-hSOD1:RFP and G85R-hSOD1:YFP.
(PDF)

Figure S19 Representative images from cells co-expressing G85R-hSOD1:RFP and A4V-hSOD1:YFP or G37R-hSOD1:YFP.
(PDF)

Figure S20 Representative images from cells co-expressing G85R-hSOD1:RFP and G85R-hSOD1:YFP.
(PDF)

Table S1 Behavior of WT and mutant hSOD1 fused to RFP or YFP in CHO cells. This table summarizes our observations of the morphology of YFP fluorescence for fusion proteins expressed in CHO cells.
(PDF)

Table S2 Behavior of WT-hSOD1:RFP or WT-hSOD1mon:RFP with WT or mutant SOD1:YPF.
(PDF)

Table S3 Behavior of WT-hSOD1:YFP and WT-hSOD1mon:YFP with mutant SOD1:RFP.
(PDF)

Table S4 Behavior of co-expressed mutant hSOD1:RFP with mutant hSOD1:YFP.
(PDF)
