# Supplementary material for: An Analysis of Interactions between Fluorescently-Tagged Mutant and Wild-Type SOD1 in Intracellular Inclusions
Source: PLoS One. 2013 Dec 31;8(12):e83981. doi: 10.1371/journal.pone.0083981 (PMC3877123; doi:10.1371/journal.pone.0083981)
Supplement: Figure S2 — Representative images from cells expressing G85RR-hSOD1:RFP or G85R-hSOD1:YFP. (PDF) [file pone.0083981.s002.pdf]

Fig. S2

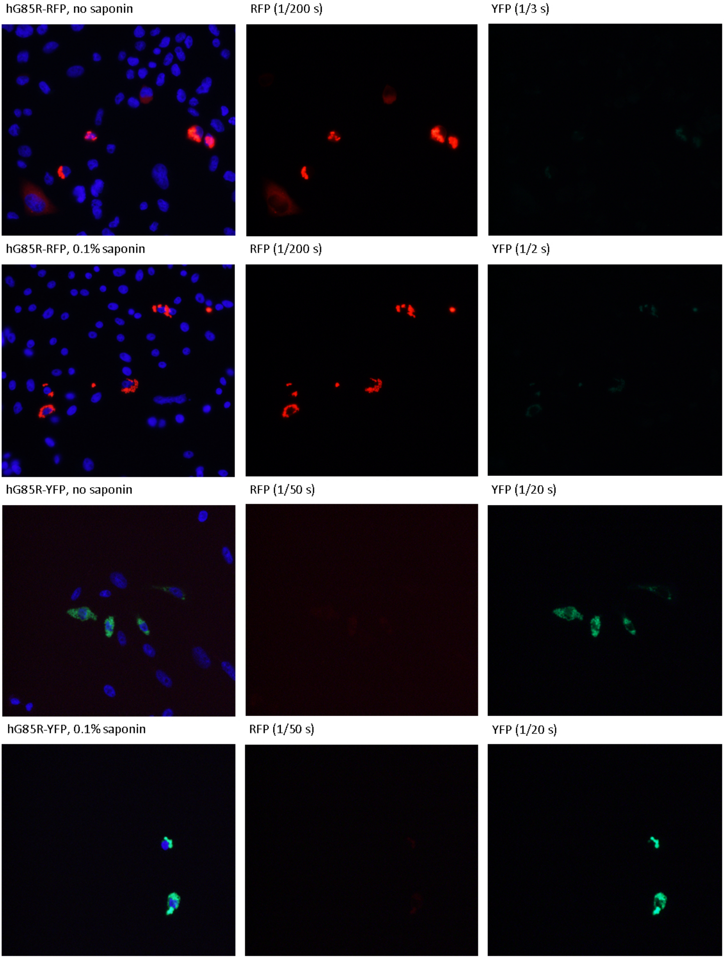
hG85R-RFP, no saponin
RFP (1/200 s)
YFP (1/3 s)
hG85R-RFP, 0.1% saponin
RFP (1/200 s)
YFP (1/2 s)
hG85R-YFP, no saponin
RFP (1/50 s)
YFP (1/20 s)
hG85R-YFP, 0.1% saponin
RFP (1/50 s)
YFP (1/20 s)
