# Supplementary figures and images for: An Analysis of Interactions between Fluorescently-Tagged Mutant and Wild-Type SOD1 in Intracellular Inclusions
Source: PLoS One. 2013 Dec 31;8(12):e83981. doi: 10.1371/journal.pone.0083981 (PMC3877123; doi:10.1371/journal.pone.0083981)

Fig. S3

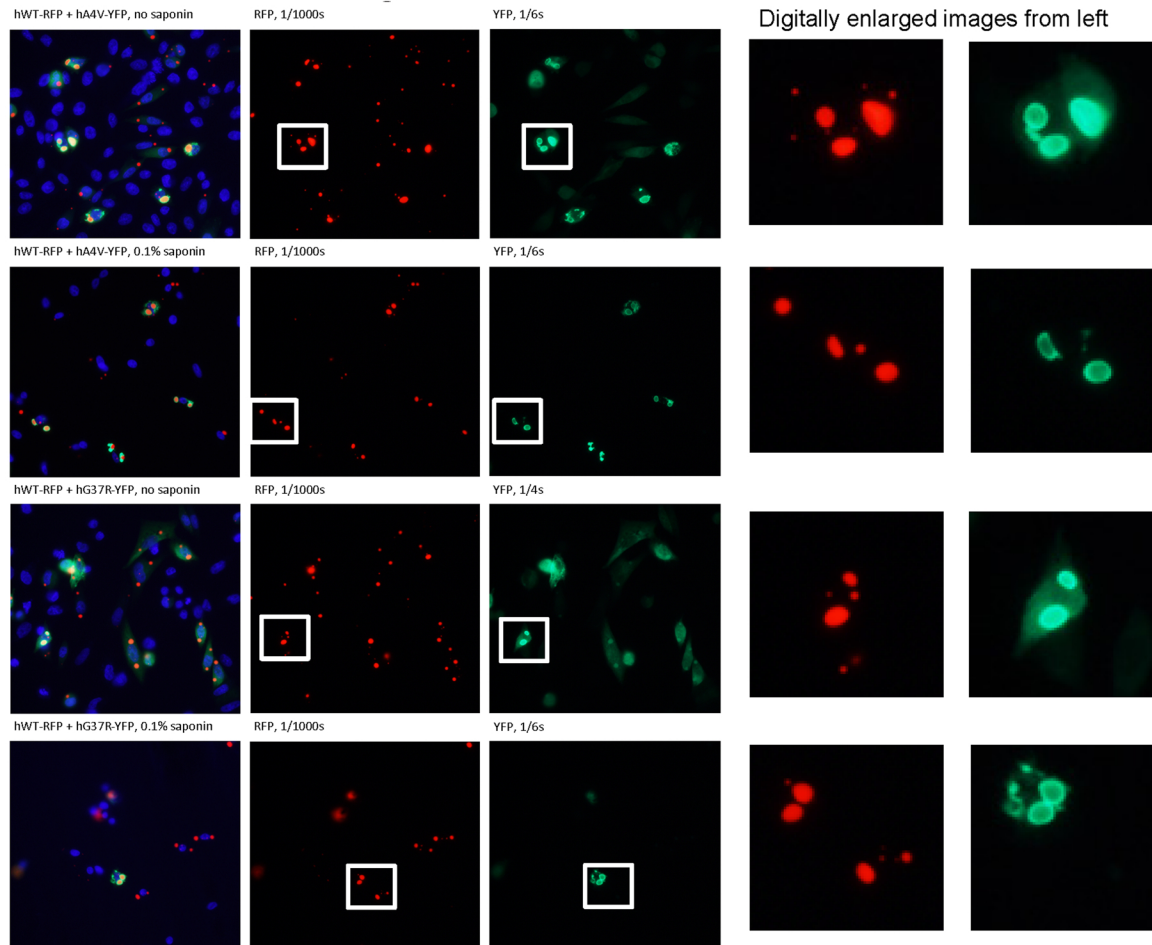

Supplement: Figure S3 — Representative images from cells co-expressing WT-hSOD1:RFP and A4V-hSOD1:YFP; and cells co-expressing WT-hSOD1:RFP and G37R-hSOD1:YFP. (PDF) [file pone.0083981.s003.pdf]

Fig. S6

hWT-RFP + hWTmon-YFP, no saponin

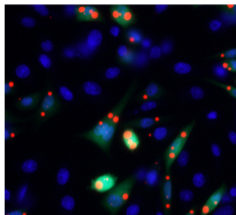

RFP (1/300 s)

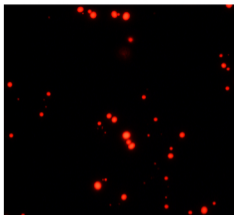

YFP, 1/2 s

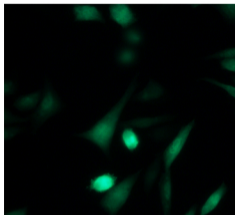

hWT-RFP + hWTmon-YFP, 0.1% saponin

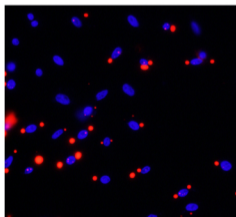

RFP (1/300 s)

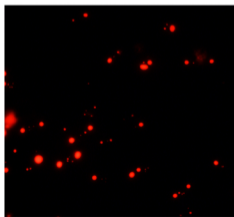

YFP, 1/2 s

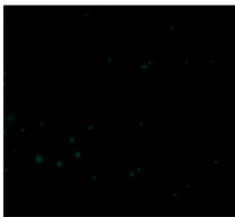

Supplement: Figure S6 — Representative images of cells co-expressing WT-hSOD1:RFP and WT-hSOD1mon:YFP. (PDF) [file pone.0083981.s006.pdf]

# Fig. S7

hWTmon-RFP + hG37R-YFP, no saponin

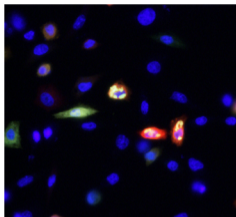

RFP (1/200 s)

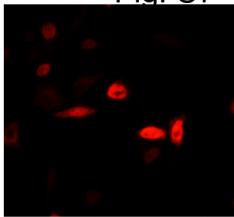

YFP (1/3.5 s)

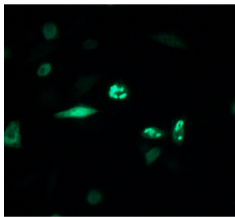

hWTmon-RFP + hG37R-YFP, 0.1% saponin

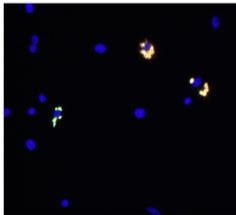

RFP (1/100 s)

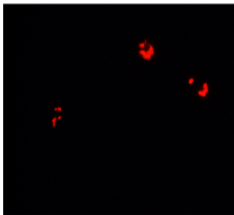

YFP (1/6 s)

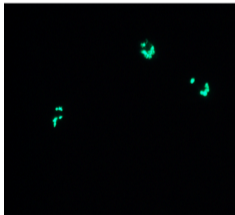

Supplement: Figure S7 — Representative images from cells co-expressing WT-hSOD1mon:RFP and G37R-hSOD1:YFP. (PDF) [file pone.0083981.s007.pdf]

Fig. S8

hWTmon-RFP + hG85R-YFP, no saponin

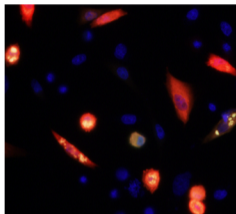

RFP (1/100 s)

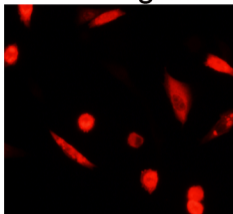

YFP (1/2 s)

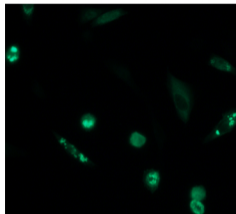

hWTmon-RFP + hG85R-YFP, 0.1% saponin

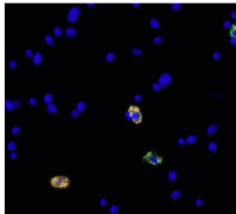

RFP (1/300 s)

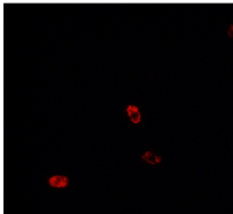

YFP (1/4 s)

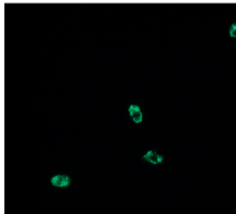

Supplement: Figure S8 — Representative images from cells co-expressing WT-hSOD1mon:RFP and G85R-hSOD1:YFP. (PDF) [file pone.0083981.s008.pdf]

Fig. S9

hA4V-RFP + hWTmon-YFP, no saponin

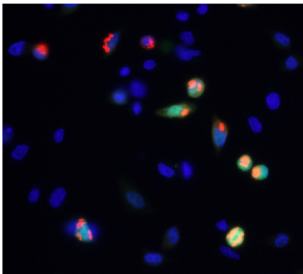

RFP (1/200 s)

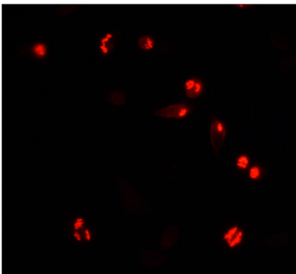

YFP (1/3 s)

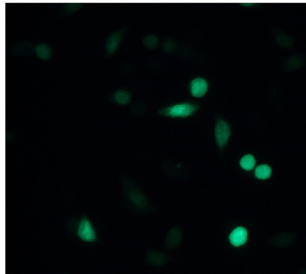

hA4V-RFP + hWTmon-YFP, 0.1% saponin

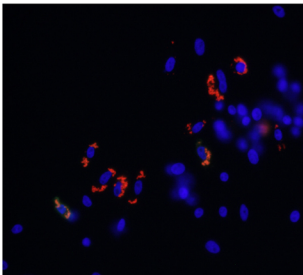

RFP (1/200 s)

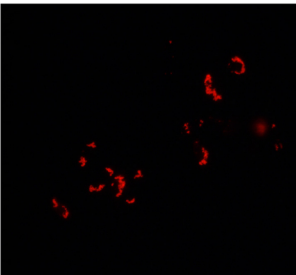

YFP (1/2 s)

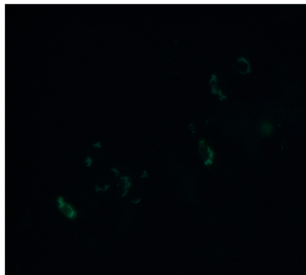

Supplement: Figure S9 — Representative images from cells co-expressing A4V-hSOD1:RFP and WT-hSOD1mon:YFP. (PDF) [file pone.0083981.s009.pdf]

Fig. S10

hG37R-RFP + hWTmon-YFP, no saponin

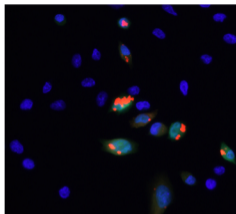

RFP (1/300 s)

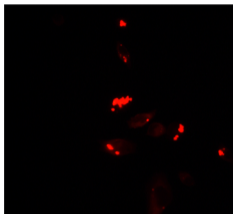

YFP (1/2 s)

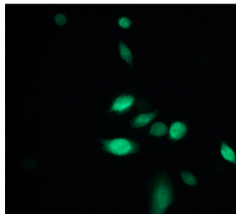

hG37R-RFP + hWTmon-YFP, 0.1% saponin

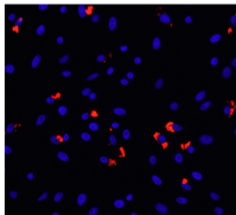

RFP (1/300 s)

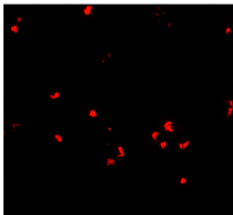

YFP (1/2 s)

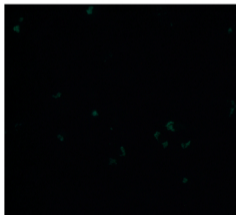

Supplement: Figure S10 — Representative images from cells co-expressing G37R-hSOD1:RFP and WT-hSOD1mon:YFP. (PDF) [file pone.0083981.s010.pdf]

Fig. S11

hG85R-RFP + hWTmon-YFP, no saponin

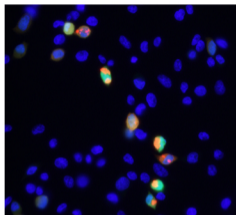

RFP (1/100 s)

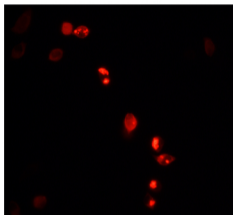

YFP (1/3 s)

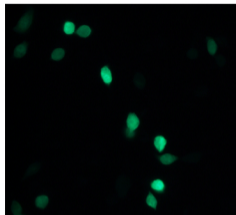

hG85R-RFP + hWTmon-YFP, 0.1% saponin

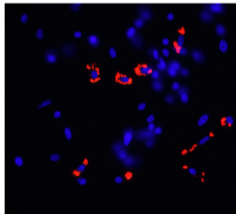

RFP (1/100 s)

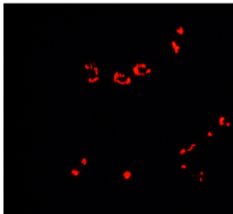

YFP (1/2 s)

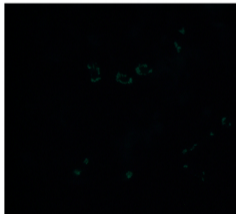

Supplement: Figure S11 — Representative images from cells co-expressing G85R-hSOD1:RFP and WT-hSOD1mon:YFP. (PDF) [file pone.0083981.s011.pdf]

# Fig. S12

hA4V-RFP + hWT-YFP, no saponin

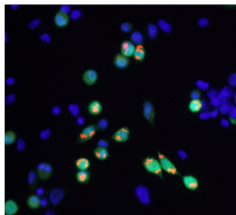

RFP (1/200 s)

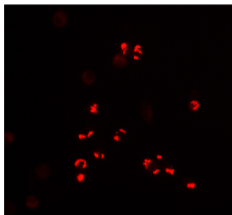

YFP (1/3 s)

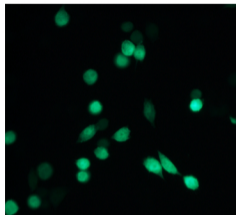

hA4V-RFP + hWT-YFP, 0.1% saponin

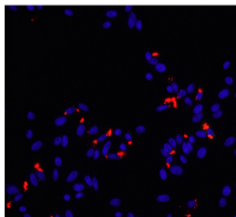

RFP (1/200 s)

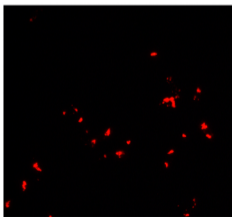

YFP (1/2 s)

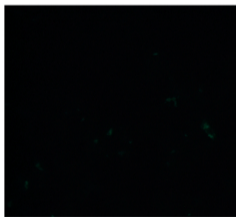

Supplement: Figure S12 — Representative images from cells co-expressing A4V-hSOD1:RFP and WT-hSOD1:YFP. (PDF) [file pone.0083981.s012.pdf]

# Fig. S13

hG37R-RFP + hWT-YFP, no saponin

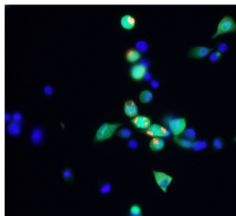

RFP (1/200 s)

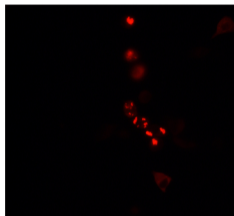

YFP (1/4 s)

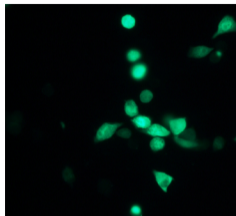

hG37R-RFP + hWT-YFP, 0.1% saponin

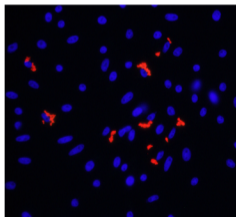

RFP (1/200 s)

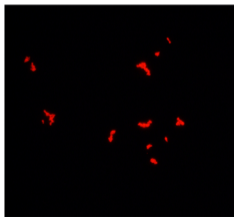

YFP (1/2 s)

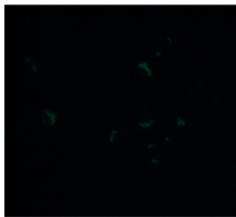

Supplement: Figure S13 — Representative images from cells co-expressing G37R-hSOD1:RFP and WT-hSOD1:YFP. (PDF) [file pone.0083981.s013.pdf]

# Fig. S14

hG85R-RFP + hWT-YFP, no saponin

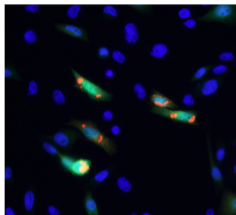

RFP (1/100 s)

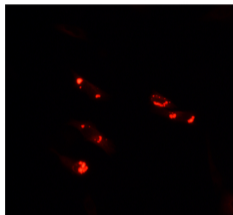

YFP (1/2 s)

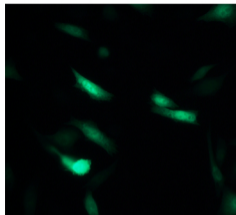

hG85R-RFP + hWT-YFP, 0.1% saponin

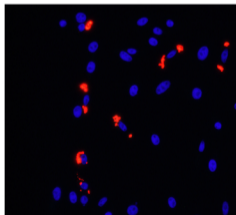

RFP (1/100 s)

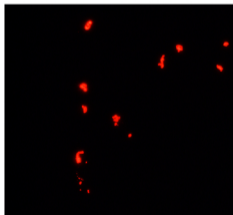

YFP (1/2 s)

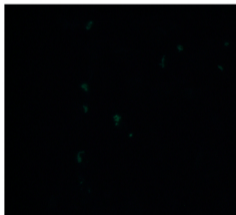

Supplement: Figure S14 — Representative images from cells co-expressing G85R-hSOD1:RFP and WT-hSOD1:YFP. (PDF) [file pone.0083981.s014.pdf]

Fig. S16

hA4V-RFP + hG85R-YFP, no saponin

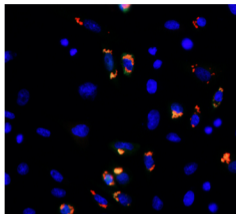

RFP (1/100 s)

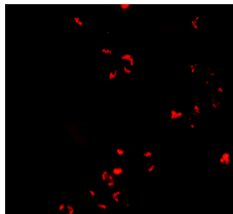

YFP (1/2 s)

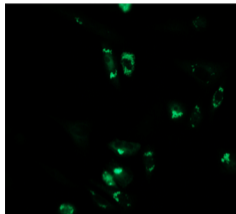

hA4V-RFP + hG85R-YFP, 0.1% saponin

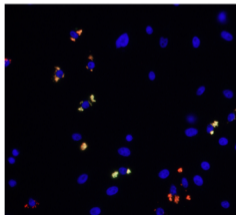

RFP (1/100 s)

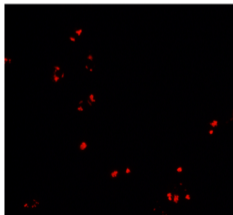

YFP (1/2 s)

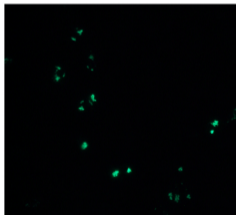

Supplement: Figure S16 — Representative images from cells co-expressing A4V-hSOD1:RFP and G85R:hSOD1:YFP. (PDF) [file pone.0083981.s016.pdf]

# Fig. S18

hG37R-RFP + hG85R-YFP, no saponin

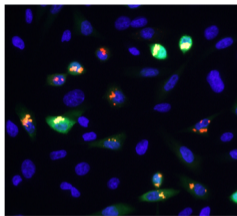

RFP (1/200 s)

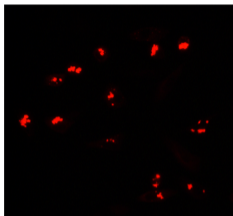

YFP (1/3 s)

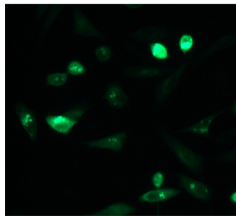

hG37R-RFP + hG85R-YFP, 0.1% saponin

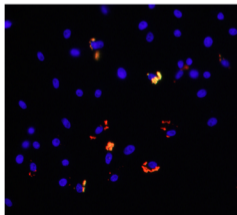

RFP (1/200 s)

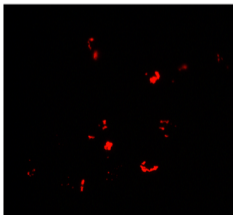

YFP (1/4 s)

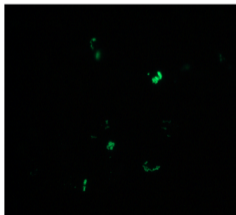

Supplement: Figure S18 — Representative images from cells co-expressing G37R-hSOD1:RFP and G85R-hSOD1:YFP. (PDF) [file pone.0083981.s018.pdf]

Fig. S20

hG85R-RFP + hG85R-YFP, no saponin

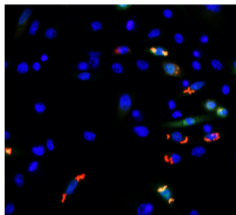

RFP (1/100 s)

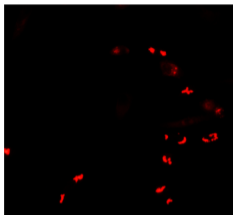

YFP (1/2 s)

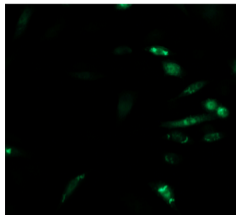

hG85R-RFP + hG85R-YFP, 0.1% saponin

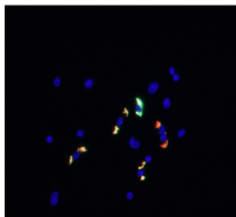

RFP (1/200 s)

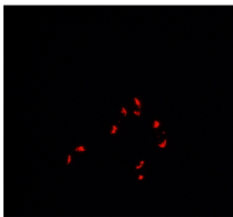

YFP (1/2 s)

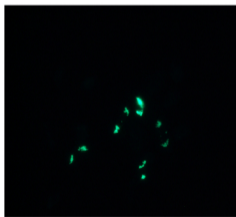

Supplement: Figure S20 — Representative images from cells co-expressing G85R-hSOD1:RFP and G85R-hSOD1:YFP. (PDF) [file pone.0083981.s020.pdf]
