# Supplementary material for: An Analysis of Interactions between Fluorescently-Tagged Mutant and Wild-Type SOD1 in Intracellular Inclusions
Source: PLoS One. 2013 Dec 31;8(12):e83981. doi: 10.1371/journal.pone.0083981 (PMC3877123; doi:10.1371/journal.pone.0083981)
Supplement: Figure S4 — Representative images from cells co-expressing WT-hSOD1:RFP and G85R-hSOD1:YFP. (PDF) [file pone.0083981.s004.pdf]

Fig. S4

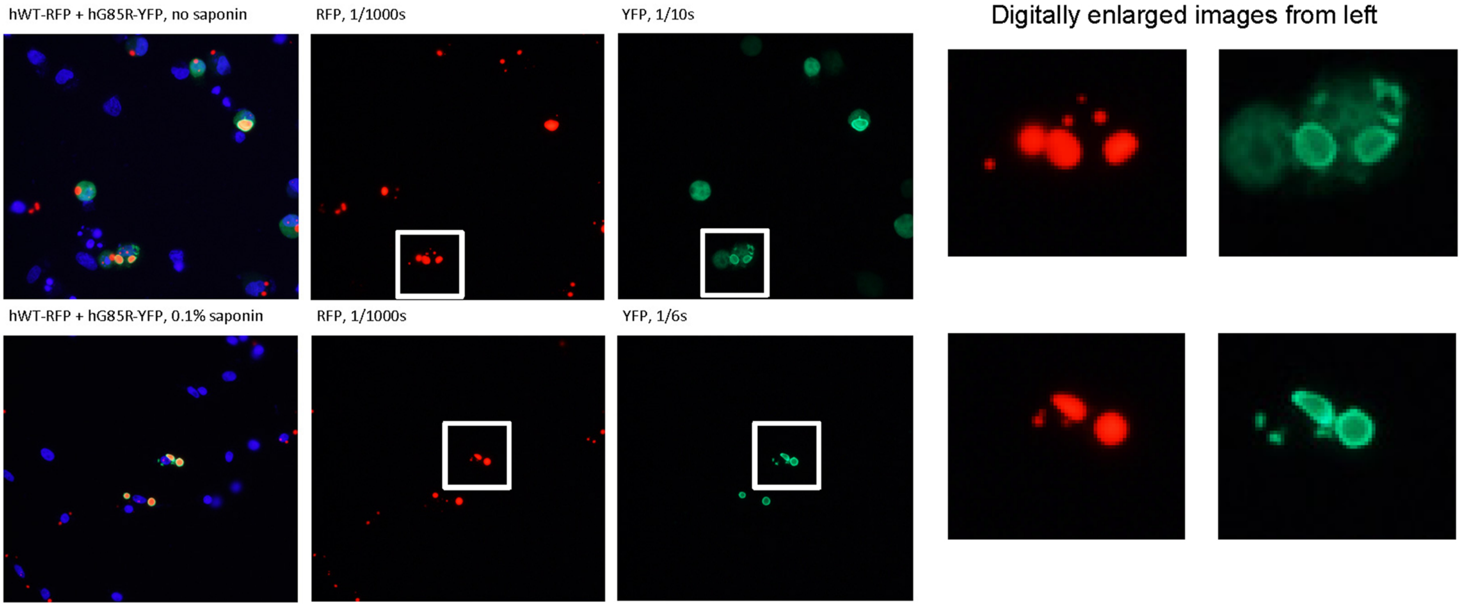
hWT-RFP + hG85R-YFP, no saponin
RFP, 1/1000s
YFP, 1/10s
Digitally enlarged images from left
hWT-RFP + hG85R-YFP, 0.1% saponin
RFP, 1/1000s
YFP, 1/6s
