# Supplementary material for: An Analysis of Interactions between Fluorescently-Tagged Mutant and Wild-Type SOD1 in Intracellular Inclusions
Source: PLoS One. 2013 Dec 31;8(12):e83981. doi: 10.1371/journal.pone.0083981 (PMC3877123; doi:10.1371/journal.pone.0083981)
Supplement: Figure S5 — Representative images from cells co-expressing WT-hSOD1mon:RFP and WT-hSOD1:YFP; and cells co-expressing WT-hSOD1mon:RFP and WT-hSOD1mon:YFP. (PDF) [file pone.0083981.s005.pdf]

Fig. S5

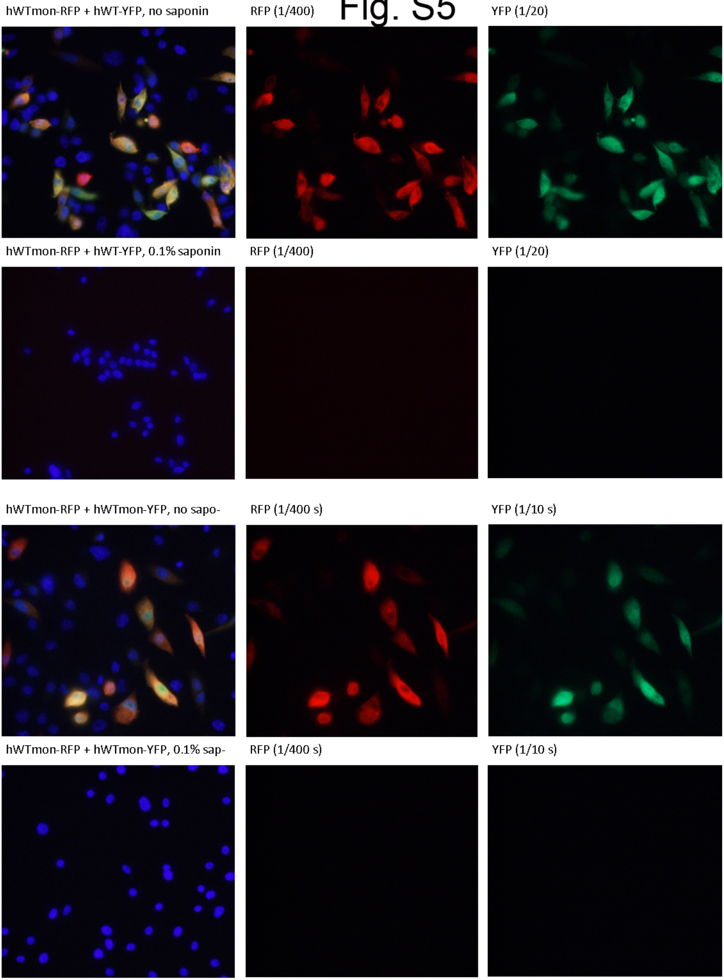
hWTmon-RFP + hWT-YFP, no saponin
RFP (1/400)
YFP (1/20)
hWTmon-RFP + hWT-YFP, 0.1% saponin
RFP (1/400)
YFP (1/20)
hWTmon-RFP + hWTmon-YFP, no sapo-
RFP (1/400 s)
YFP (1/10 s)
hWTmon-RFP + hWTmon-YFP, 0.1% sap-
RFP (1/400 s)
YFP (1/10 s)
