# Supplementary material for: An Analysis of Interactions between Fluorescently-Tagged Mutant and Wild-Type SOD1 in Intracellular Inclusions
Source: PLoS One. 2013 Dec 31;8(12):e83981. doi: 10.1371/journal.pone.0083981 (PMC3877123; doi:10.1371/journal.pone.0083981)
Supplement: Figure S19 — Representative images from cells co-expressing G85R-hSOD1:RFP and A4V-hSOD1:YFP or G37R-hSOD1:YFP. (PDF) [file pone.0083981.s019.pdf]

Fig. S19

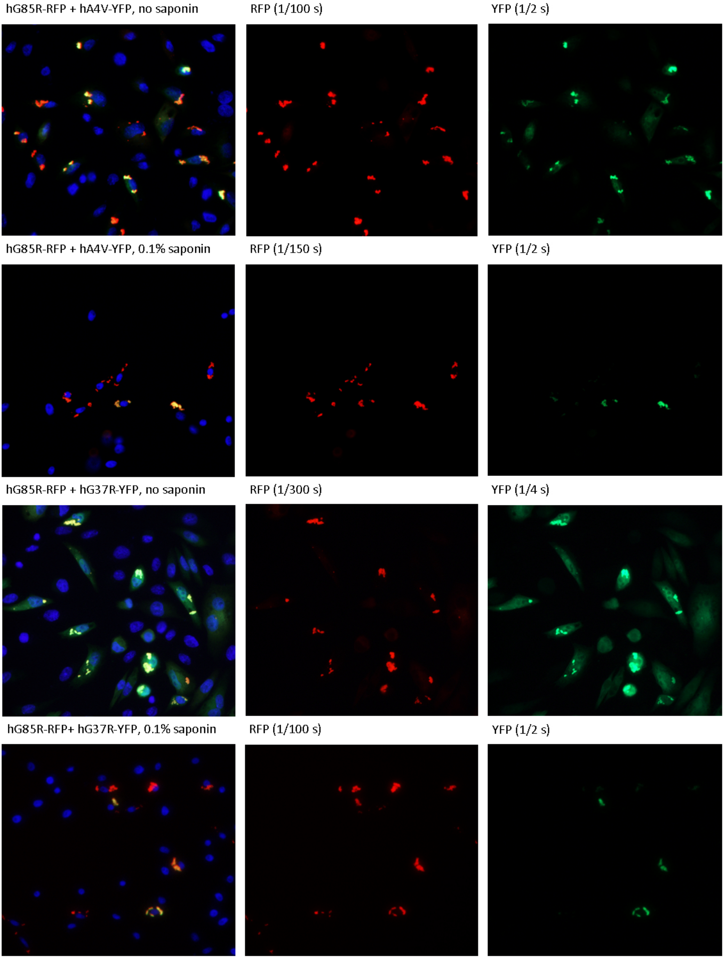

hG85R-RFP + hA4V-YFP, no saponin
RFP (1/100 s)
YFP (1/2 s)
hG85R-RFP + hA4V-YFP, 0.1% saponin
RFP (1/150 s)
YFP (1/2 s)
hG85R-RFP + hG37R-YFP, no saponin
RFP (1/300 s)
YFP (1/4 s)
hG85R-RFP+ hG37R-YFP, 0.1% saponin
RFP (1/100 s)
YFP (1/2 s)
