# Supplementary material for: An Analysis of Interactions between Fluorescently-Tagged Mutant and Wild-Type SOD1 in Intracellular Inclusions
Source: PLoS One. 2013 Dec 31;8(12):e83981. doi: 10.1371/journal.pone.0083981 (PMC3877123; doi:10.1371/journal.pone.0083981)
Supplement: Table S1 — Behavior of WT and mutant hSOD1 fused to RFP or YFP in CHO cells. This table summarizes our observations of the morphology of YFP fluorescence for fusion proteins expressed in CHO cells. (PDF) [file pone.0083981.s021.pdf]

**Table S1. Behavior of WT and mutant hSOD1 fused to RFP or YFP in CHO cells.**

| Gene Expressed | Forms inclusions? | Saponin-resistant | Morphology | Additional Reference |
|---|---|---|---|---|
| YFP | No | No* | N/A | [4] |
| WT-hSOD1:YFP | No | No | N/A | [4] |
| WT-hSOD1mon:YFP | No | No | N/A | |
| A4V-hSOD1:YFP | Yes | Yes | Varigated | [6] |
| G37R-hSOD1:YFP | Yes | Yes | Varigated | |
| G85R-hSOD1:YFP | Yes | Yes | Varigated | |
| | | | | |
| RFP | No | No | N/A | [4] |
| WT-hSOD1:RFP | Yes | Yes | Round | [4] |
| WT-hSOD1mon:RFP | No | No | N/A | |
| A4V-hSOD1:RFP | Yes | Yes | Varigated | [4] |
| G37R-hSOD1:RFP | Yes | Yes | Varigated | |
| G85R-hSOD1:RFP | Yes | Yes | Varigated | |

*A fraction of YFP diffuses into the nucleus and does not diffuse out with saponin treatment.
