# Supplementary material for: An Analysis of Interactions between Fluorescently-Tagged Mutant and Wild-Type SOD1 in Intracellular Inclusions
Source: PLoS One. 2013 Dec 31;8(12):e83981. doi: 10.1371/journal.pone.0083981 (PMC3877123; doi:10.1371/journal.pone.0083981)
Supplement: Table S2 — Behavior of WT-hSOD1:RFP or WT-hSOD1mon:RFP with WT or mutant SOD1:YPF. (PDF) [file pone.0083981.s022.pdf]

Table S2. Behavior of WT-hSOD1:RFP or WT-hSOD1mon:RFP with WT or mutant SOD1:YFP.

Genes Expressed	Forms inclusions?	Intermingled or Layered?	Morphology	Saponin-resistant
WT-hSOD1:RFP<br>WT-hSOD1:YFP	Yes<br>Yes	Intermingled	Round	Yes<br>No
WT-hSOD1:RFP<br>WT-hSOD1mon:YFP	Yes<br>No	N/A	Round	Yes<br>No
WT-hSOD1:RFP<br>A4V-hSOD1:YFP	Yes<br>Yes	Layered	Round	Yes<br>Yes
WT-hSOD1:RFP<br>G37R-hSOD1:YFP	Yes<br>Yes	Layered	Round	Yes<br>Yes
WT-hSOD1:RFP<br>G85R-hSOD1:YFP	Yes<br>Yes	Layered	Round	Yes<br>Yes
WT-hSOD1mon:RFP<br>WT-hSOD1mon:YFP	No<br>No	N/A		Yes<br>Yes
WT-hSOD1mon:RFP<br>A4V-hSOD1:YFP	Yes<br>Yes	Intermingled	Varigated	Yes<br>Yes
WT-hSOD1mon:RFP<br>G37R-hSOD1:YFP	Yes<br>Yes	Intermingled	Varigated	Yes<br>Yes
WT-hSOD1mon:RFP<br>G85R-hSOD1:YFP	Yes<br>Yes	Intermingled	Varigated	Yes<br>Yes
