# Supplementary material for: An Analysis of Interactions between Fluorescently-Tagged Mutant and Wild-Type SOD1 in Intracellular Inclusions
Source: PLoS One. 2013 Dec 31;8(12):e83981. doi: 10.1371/journal.pone.0083981 (PMC3877123; doi:10.1371/journal.pone.0083981)
Supplement: Table S3 — Behavior of WT-hSOD1:YFP and WT-hSOD1mon:YFP with mutant SOD1:RFP. (PDF) [file pone.0083981.s023.pdf]

Table S3. Behavior of WT-hSOD1:YFP and WT-hSOD1mon:YFP with mutant SOD1:RFP

Genes Expressed	Forms inclusions?	Intermingled or Layered?	Morphology	Saponin-resistant
WT-hSOD1:YFP<br>A4V-hSOD1:RFP	No<br>Yes	N/A	Varigated	No<br>Yes
WT-hSOD1:YFP<br>G37R-hSOD1:RFP	No<br>Yes	N/A	Varigated	No<br>Yes
WT-hSOD1:YFP<br>G85R-hSOD1:RFP	No<br>Yes	N/A	Varigated	No<br>Yes
WT-hSOD1mon:YFP<br>A4V-hSOD1:RFP	No<br>Yes	N/A	Varigated	No<br>Yes
WT-hSOD1mon:YFP<br>G37R-hSOD1:RFP	No<br>Yes	N/A	Varigated	No<br>Yes
WT-hSOD1mon:YFP<br>G85R-hSOD1:RFP	No<br>Yes	N/A	Varigated	No<br>Yes
