# Supplementary material for: An Analysis of Interactions between Fluorescently-Tagged Mutant and Wild-Type SOD1 in Intracellular Inclusions
Source: PLoS One. 2013 Dec 31;8(12):e83981. doi: 10.1371/journal.pone.0083981 (PMC3877123; doi:10.1371/journal.pone.0083981)
Supplement: Table S4 — Behavior of co-expressed mutant hSOD1:RFP with mutant hSOD1:YFP. (PDF) [file pone.0083981.s024.pdf]

Table S4. Behavior of co-expressed mutant hSOD1:RFP with mutant hSOD1:YFP.

Genes Expressed	Forms inclusions?	Intermingled or Layered?	Morphology	Saponin-resistant
A4V-hSOD1:RFP<br>A4V-hSOD1:YFP	Yes<br>Yes	Intermingled	Varigated	Yes<br>Yes
A4V-hSOD1:RFP<br>G37R-hSOD1:YFP	Yes<br>Yes	Intermingled	Varigated	Yes<br>Yes
A4V-hSOD1:RFP<br>G85R-hSOD1:YFP	Yes<br>Yes	Intermingled	Varigated	Yes<br>Yes
G37R-hSOD1:RFP<br>A4V-hSOD1:YFP	Yes<br>Yes	Intermingled	Varigated	Yes<br>Yes
G37R-hSOD1:RFP<br>G37R-hSOD1:YFP	Yes<br>Yes	Intermingled	Varigated	Yes<br>Yes
G37R-hSOD1:RFP<br>G85R-hSOD1:YFP	Yes<br>Yes	Intermingled	Varigated	Yes<br>Yes
G85R-hSOD1:RFP<br>A4V-hSOD1:YFP	Yes<br>Yes	Intermingled	Varigated	Yes<br>Yes
G85R-hSOD1:RFP<br>G37R-hSOD1:YFP	Yes<br>Yes	Intermingled	Varigated	Yes<br>Yes
G85R-hSOD1:RFP<br>G85R-hSOD1:YFP	Yes<br>Yes	Intermingled	Varigated	Yes<br>Yes
